# Fabrication of macroporous reduced graphene oxide composite aerogels reinforced with chitosan for high bilirubin adsorption

**DOI:** 10.1039/c8ra00358k

**Published:** 2018-02-22

**Authors:** Zhentao Li, Xi Song, Siyuan Cui, Yanpeng Jiao, Changren Zhou

**Affiliations:** Department of Materials Science and Engineering, Jinan University Guangzhou 510632 China tjiaoyp@jnu.edu.cn +86-20-85223271 +86-20-85223271

## Abstract

Numerous adsorbents have been reported for the removal of bilirubin, which is a well-known endogenous toxin. Three-dimensional graphene sponges and aerogels have been fully studied in the adsorption field but little in hemoperfusion especially for bilirubin adsorption. In this study, macroporous reduced graphene oxide (GO) aerogels were fabricated by a chemical reduction method. Besides, chitosan was introduced in the aerogels during the reduction process to improve their mechanical properties. The graphene oxide composite aerogels reinforced with chitosan (rGO/CS) were investigated using scanning electron microscopy (SEM), Fourier Transform Infrared Spectrometry (FTIR), Raman spectroscopy, X-ray photoelectron spectroscopy (XPS), X-ray diffraction (XRD) and Brunauer–Emmett–Teller (BET). Furthermore, the mechanical properties test showed the reinforced mechanical strength of the rGO/CS aerogels. The adsorption performance of the aerogels for bilirubin was studied in detail, showing a high adsorption capacity (458.9 mg g^−1^) and a fast adsorption rate. Moreover, the low hemolysis ratio and negligible anticoagulant activity of rGO/CS aerogels suggested good blood compatibility. The mesoporous structure of the aerogels can provide good mechanical strength, and the macroporous structure of the rGO/CS aerogels shows a good adsorption capacity, which would have potential applications in bilirubin adsorption.

## Abstract 2

Chitosan reinforced macroporous reduced graphene oxide composite aerogels were fabricated with high bilirubin adsorption properties.

## Graphical abstract

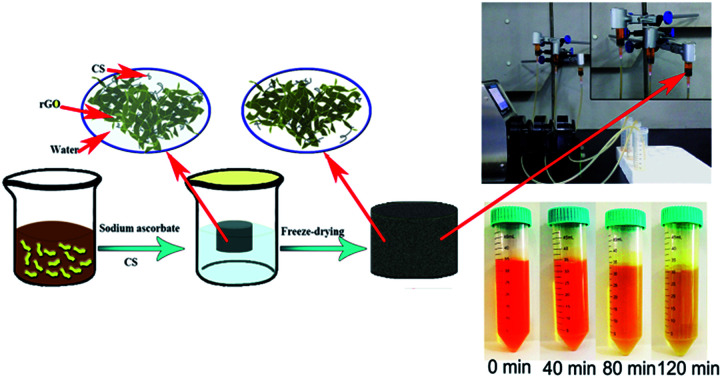

## 1. Introduction

**Fig. 1:**
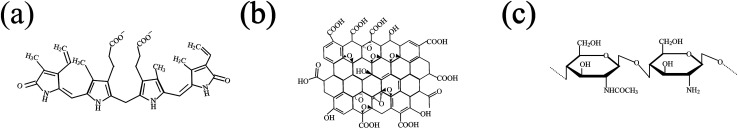
Bilirubin molecular structure (a); graphene oxide molecular structure (b); chitosan molecular formula (c).

Bilirubin (Fig. 1(a)), one of the breakdown products of red blood cell degradation,^1^ is a typical toxin in the blood.^2^ Hyperbilirubinemia can cause brain damage in neonates,^3^ resulting in hepatitis, jaundice, and even death in severe cases.^4^ Compared with phototherapy, haemodialysis and exchange blood transfusion methodologies, haemoperfusion has become a better choice for treating hyperbilirubinemia.^5,6^ At present, various adsorbents, such as resins, porous silicon materials, porous carbon, TiO_2_, polymer fiber membranes and bilirubin-imprinted particles, have been used for bilirubin adsorption.^7–12^ Ideal adsorbents should possess high adsorption capacity, sufficient mechanical strength and good biocompatibility, which is still a challenge for us.

Graphene has excellent physical and chemical properties and has been widely applied to various adsorption materials.^13–15^ In some practical applications, graphene is usually packaged in a three-dimensional (3D) structure.^16^ The preparation of mesoporous graphene aerogels by graphene oxide (GO, see Fig. 1(b))^17,18^ can be realized by the strong reductant and hydrothermal method.^19,20^ In addition, 3D porous graphene composites have showed some new properties, such as high compressibility, ultra low density and strong mechanical strength,^21^ suggesting a good candidate to be adsorbents.^13,22^ There are some reports on graphene as functional additives to fabricate polymer and graphene composites for bilirubin adsorption,^5,23^ but few research on bilirubin adsorption by graphene used as a main matrix material.

Usually, porosity is regarded as an inversely proportional influence factor to the strength of the graphene aerogels. The enhanced strength of graphene aerogels would inevitably lead to a decrease of their porosity,^24^ which is crucial to the capacity of adsorption. To prepare a 3D graphene aerogels with good mechanical strength and high porosity, it is feasible to enhance the strength of the hole structure of the graphene aerogels by decorating other polymer materials,^25^ including chitosan.^26,27^

Chitosan (CS, see in Fig. 1(c)), with good biocompatibility, biodegradation and antifungal activity, had been extensively used in biomedical fields^28,29^ including bilirubin adsorption.^30^ CS can crosslink with GO sheets by the –NH_2_ groups and hydrogen-bond interaction. During the reduction process of GO, CS can drag the graphene sheets to complete the self-assembly process.^23,31^ This process of self-assembly could form a porous structure with high mechanical strength, which could satisfy the requirement bilirubin adsorption.

In this study, rGO-based composite aerogels were prepare by the self-assembly of graphene using sodium ascorbate as a reducing agent. The addition of CS ensured that the rGO-based composite aerogels had a good pore structure and excellent mechanical strength. Simultaneously, embedding CS was an effective way to avoid the defects of other preparation methods, like toxicity and complex synthetic route. The study demonstrated that the rGO-based composite aerogels had high adsorption capacity for bilirubin with good mechanical properties, as well as good blood compatibility.

## 2. Materials and methods

### 2.1 Materials

High purity graphite flake (300 meshes) was obtained from Nanjing Xianfeng Nano Materials Technology Co., Ltd. (Nanjing, China). Bilirubin was bought from Macklin Biochemical Technology Co., Ltd (Shanghai, China). CS (>95% degree of deacetylation) and sodium ascorbate (99%) were obtained from Aladdin Biochemical Technology Co., Ltd. (Shanghai, China). Other experimental materials, such as the sulfuric acid, potassium permanganate, hydrochloric acid and hydrogen peroxide (30%, w/w) were obtained from Beijing Chemical Reagents Company (China).

### 2.2 Preparation of rGO/CS*n* aerogels

The GO was obtained from high purity flake graphite through the oxidation process according to the modified Hummers' method.^19,32^ The different concentration of CS solutions (0.2, 0.4 and 0.8 mg mL^−1^) were prepared by dissolving CS in a 2.5% (v/v) acetic acid aqueous. GO suspension (4 mg mL^−1^) was prepared by dispersing GO in distilled water under sonicating. Then, the prepared GO suspension (3 mL) was mixed with corresponding CS solution (3 mL) containing sodium ascorbate (40 mg mL^−1^) by stirring for 10 min. And the control one was prepared with the equal volume of aqueous acetic acid (2.5% v/v) and sodium ascorbate instead of CS solution under the same condition. Then the resulting mixture was kept under 60 °C for 6 hours to get the rGO/CS*n* hydrogels, which were further washed with distilled water to remove excessive acid. After freeze drying, rGO/CS aerogels were obtained. In the study, the rGO/CS aerogels were labelled as “rGO/CS*n*”, and the “*n*” means the ratio of CS, which were rGO/CS0 (100 : 0, w/w), rGO/CS5 (100 : 5, w/w), rGO/CS10 (100 : 10, w/w) and rGO/CS20 (100 : 20, w/w), respectively.

### 2.3 Characterization of rGO/CS*n* aerogels

The rGO/CS*n* aerogels were coated with gold and observed with a scanning electron microscope (SEM, XL30 FESEM, PHILIPS) at 20 kV accelerating voltage. A fourier transformation infrared spectroscope (FTIR, Bruker EQUINOX 55, Germany) was used to study the composition of rGO/CS*n* aerogels. The structure of the samples were analyzed by a Raman spectroscope (Alpha300R, Germany) using a 532 nm laser as the excitation source in the range of 800–3000 cm^−1^. X-ray diffraction (XRD) measurement was conducted by an X-ray diffractometer (Bruker, Germany) with Cu Kα radiation (40 kV, 20 mA, *λ* = 1.5418 Å). And the scanning angle was set in the range of 2–70° and the scanning rate was 10° min^−1^. The surface of rGO/CS*n* was investigated by an X-ray photoelectron spectroscope (XPS, ESCALAB 250, Thermo Scientific) with a monochromated Al/Kα (*hν* = 1486.6 eV) as X-ray source. The Multipak software provided by manufacturers was used to analysis the date. The Brunauer–Emmett–Teller (BET) surface area, pore volume, and pore width of the samples were characterized by nitrogen adsorption and desorption using a surface area and porosity analyzer (Micromeritics, ASAP 2460).

The mechanical properties were tested by a universal test machine. During measurement, the height and the diameter of each cylinder sample were measured with a vernier caliper. The loading and unloading speed was 2 mm min^−1^. The sample was compressed to 50% of the set stroke and then back to the starting position for a next cycle. The number of compression cycles was set to 5 times. And the apparent density of rGO/CS*n* was calculated with the weight divided by the volume.

### 2.4 Adsorption experiments

A peristaltic pump was used to simulate the process of perfusion device in the experiment to test the adsorption capacity of rGO/CS*n* for bilirubin. And the color of the bilirubin solution became weak quickly after adsorption, providing a direct visual impression of the good adsorption capacity of rGO/CS*n* aerogels. Bilirubin was dissolved in a 0.1 mol L^−1^ NaOH solution, and diluted with phosphate buffer (pH = 7.4). Perfusion experiment was conducted by 35 mL bilirubin solution (200 mg L^−1^) passing through the rGO/CS*n* aerogels (10 mg) at the room temperature in a dark environment. And the solid–liquid ratio was set to 2 : 7. The flow rate of the bilirubin solution was set 1 mL min^−1^. The equilibrium adsorption capacity (*q*_e_) (mg g^−1^) of rGO/CS*n* aerogels for bilirubin was obtained after 280 min. Moreover, the bilirubin concentration was detected by an UV-vis spectrophotometer (Shimadzu Corporation, Japan) at 438 nm.

The bilirubin adsorption capacity by rGO/CS*n* was determined with following eqn (1):1
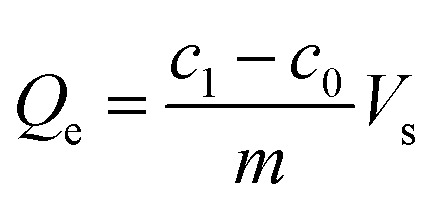
where the *Q*_e_ (mg g^−1^) is defined as the bilirubin adsorption capacity by rGO/CS*n*; *c*_0_ (mg L^−1^) and *c*_1_ (mg L^−1^) represent the initial and the final concentration of bilirubin solution, respectively; *V*_s_ (L) is the volume of bilirubin solution; *m* (g) is the mass of rGO/CS*n*.

The ratio of adsorbed bilirubin to the total bilirubin was defined as the adsorption efficiency to display the adsorption capacity of materials. The adsorption efficiency of bilirubin was determined with following eqn (2):2
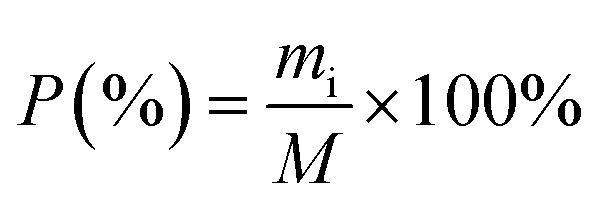
where the *P* is the adsorption efficiency of bilirubin (%), *m*_i_ is defined as the mass of bilirubin adsorbed by the rGO/CS*n*, *M* is the total mass of bilirubin in the solution.

#### 2.4.1 Adsorption kinetics

The adsorption kinetic was investigated to study the adsorption process of rGO/CS*n* for bilirubin. In addition, pseudo first-order and pseudo second-order kinetic models were used to analyze the adsorption kinetic behavior by eqn (3) and (4), respectively.

3 ln(*q*_e_ − *q*_*t*_) = ln *q*_e_ − *k*_1_*t*

4
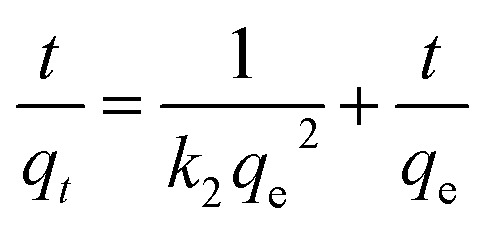
where *q*_e_ (mg g^−1^) is the calculated from saturation, *q*_*t*_ (mg g^−1^) is the adsorption amount, *t* (min) is the adsorption time, and *k*_1_ is the related rate constant of the pseudo first-order kinetic model. The value of *k*_2_ is the adsorption rate constant of pseudo-second-order model.

#### 2.4.2 Adsorption isotherm

The solid–liquid ratio of materials to the bilirubin solution remained 2 : 7, the bilirubin concentration ranged from 100 to 600 mg L^−1^, and the adsorption time was 280 min. In addition, the adsorption isotherms were fitted with Langmuir model and Freundlich mode, respectively. The Langmuir model could describe the monolayer adsorption based on the assumption of adsorption homogeneity.^33^ The Freundlich model was empirical, which was assumed the adsorption process take place on a heterogeneous surface. Moreover, the Freundlich model described the multilayer adsorption. The calculation formulas of two models were following eqn (5) and (6), respectively.5
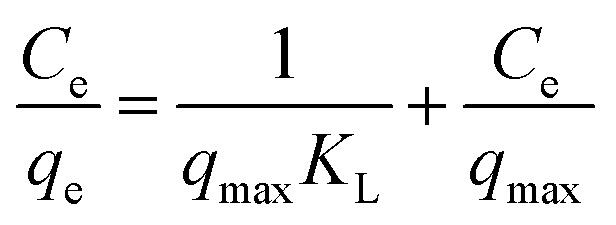
6
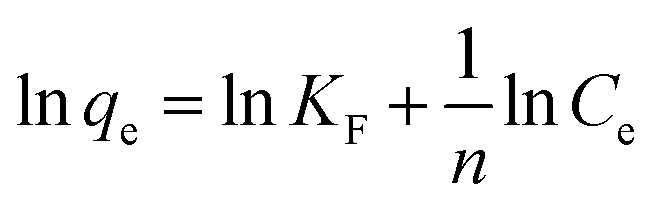
where *C*_e_ (mg L^−1^) represents the bilirubin concentration after adsorption, *q*_e_ (mg g^−1^) is the adsorption capacity for bilirubin, and *q*_max_ (mg g^−1^) is the maximal adsorbed capacity for bilirubin. *K*_L_ is the Langmuir adsorption constant, and *K*_F_ is the Freundlich adsorption constant. *n* is a Freundlich linearity index, 1/*n* is a constant indicating adsorption intensity.

#### 2.4.3 Bilirubin adsorption in the presence of BSA

The concentration of the mixed solution of albumin and bilirubin was both set as 200 mg L^−1^, respectively. Others adsorption condition was as same as the adsorption kinetics.

### 2.5 Coagulation assay and hemolysis assay

Coagulation assay and hemolysis test were preliminary assessment to the blood compatibility of biomaterials. All the human blood-related experiments were performed in accordance with the Guidelines for Care and Use of Laboratory Animals of Jinan University (Guangzhou, China) and approved by the Ethics Committee of Jinan University (Guangzhou, China). Informed consents were obtained from human participants of this study. The freshly collected sodium citrate anticoagulated whole blood was centrifuged at 3000 rpm for 10 min to isolate upper human platelet poor plasma (PPP). rGO/CS*n* (0.5 mg) were incubated in PPP (500 μL) at 37 °C for 30 min. Then the supernatant liquor (100 μL) was collected to detect the values of activated partial thromboplastin time (APTT) and prothrombin time (PT) with an automatic coagulation analyzer. Furthermore, the fresh human PPP was used as a control group during the experiment.

The hemolysis ratio was used to evaluate the destructive effect of biomaterials on the erythrocyte. In the experiment, phosphate buffer (pH = 7.4) and deionized water were used as the negative and positive control, respectively. Firstly, freshly sodium citrate anticoagulated whole blood was centrifuged at 3000 rpm for 10 min and then removed the upper plasma and pale yellow part. The centrifuged red blood cells were washed with phosphate buffer for 3 times. The rGO/CS*n* (0.5 mg) was incubated in phosphate buffer (2 mL) at 37 °C for 2 h. Then, the materials were mixed with the 2% erythrocyte suspension and incubated at 37 °C for 2 h. Then, the mixtures were centrifuged at 2000 rpm for 10 min. The absorbance of the supernatant was tested at 545 nm by a microplate reader (Multiskan MK3, Thermo Scientific). The hemolysis rate was calculated by eqn (7):7
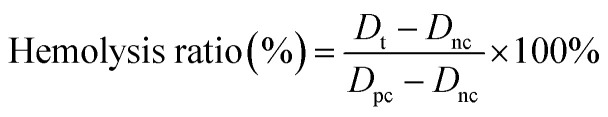
where *D*_t_, *D*_nc_, and *D*_pc_ were the absorbance values of the samples, the negative control, and the positive control, respectively.

## 3. Results and discussion

### 3.1 Characterization of GO

**Fig. 2:**
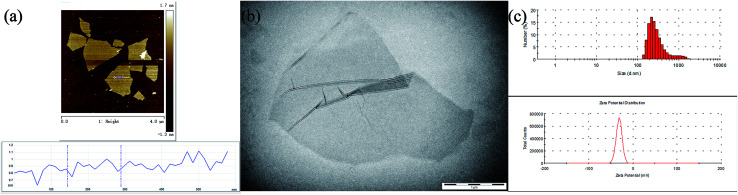
The observation of the GO morphology by AFM (a); TEM image of GO (b); the histogram of the size dispersion and zeta potential of GO nanosheets (c).

In the present study, the obtained GO could be entirely exfoliated into single-layer GO nanosheets and the thickness was about 1.0 nm (see Fig. 2(a)). It was also observed that the transverse size of the prepared monolayer GO was about 1.0 μm by transmission electron microscopy (see Fig. 2(b)). The obtained GO solution had an average particle size of about 379.0 nm and the resulting zeta potential was −29.6 mV (see Fig. 2(c)).

### 3.2 Photograph and SEM of rGO/CS*n*

**Fig. 3:**
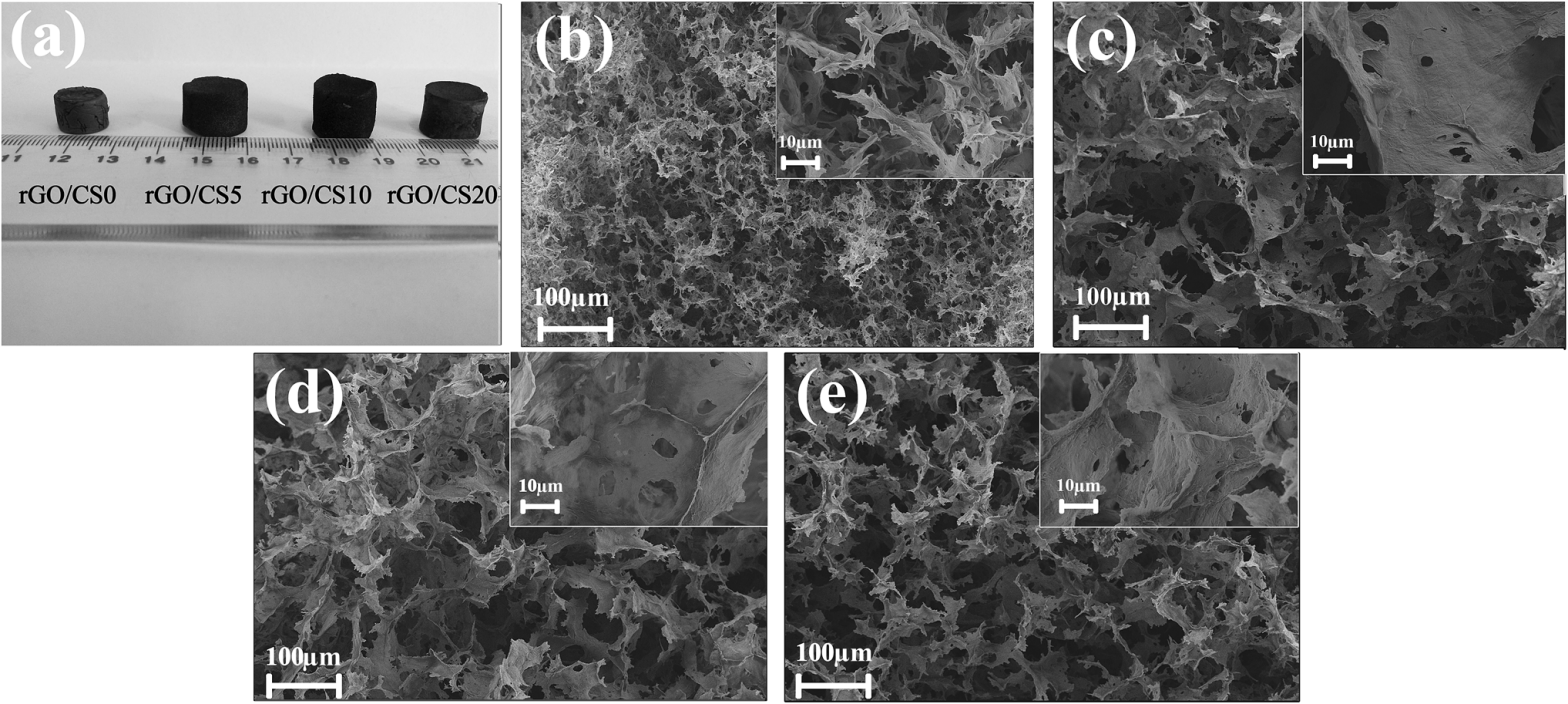
Photograph of rGO/CS*n* aerogels (a), SEM images for the rGO/CS*n* aerogels: rGO/CS0 (b), rGO/CS5 (c), rGO/CS10 (d), rGO/CS20 (e).

Fig. 3(a) showed the photographs of rGO/CS*n* aerogels, and the density of the samples were 15.06 (rGO/CS0), 5.63 (rGO/CS5), 7.02 (rGO/CS10) and 9.33 mg cm^−3^ (rGO/CS20), respectively. SEM images of rGO/CS*n* aerogels were depicted in Fig. 3(b)–(e). The rGO/CS*n* showed the analogous 3D network structure, which was built by 2D rGO sheets. And the pore sizes of the rGO/CS*n* aerogels ranged from several micrometers to tens of micrometers. Compared with the rGO/CS0, the addition of chitosan resulted in a significant reduction of the density. As showed in Fig. 3(b), the pore size of rGO/CS0 was smaller than others, and the pores walls were discontinuous and fracture. Reversely, the pores walls of composite materials were more continuous and complete as see in Fig. 3(c)–(e). The interconnected pores could improve the solute diffusion throughout the aerogels, which was crucial for adsorption applications.

The GO sheets could be easily dispersed in water during the preparation process because of the electrostatic repulsion between the sheets.^31^ The CS molecules could also be incorporated to facilitate the gelation process of GO sheets under the condition of ascorbic acid sodium.^23^ The main driving forces that was used to fabricate 3D network-like nanostructure are various weak interactions between building blocks, such as hydrogen bonding and π–π stacking.^23,34,35^ CS acted as an efficient crosslinking reagent, which contributed to porous structure of the rGO/CS*n* aerogels. Besides, the multiple hydrogen bonds between CS chains and GO sheets also could form, which could increase the bonding force among GO sheets.^31^

### 3.3 Structure and component analysis of rGO/CS*n*

#### 3.3.1 FTIR analysis of rGO/CS*n*

**Fig. 4:**
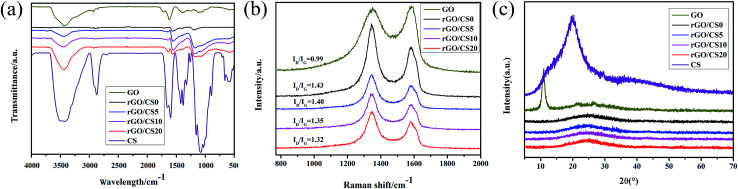
FT-IR spectra (a), Raman spectroscopy (b) and XRD patterns (c) of GO and rGO/CSn samples.

The FTIR spectra of the samples were showed in Fig. 4(a). For the spectrum of GO sheets, the characteristic peaks at 3424 and 1389 cm^−1^ could be attributed to the –OH stretching vibration and bending vibration. In addition, other obvious peaks, such as 1730, 1226 and 1062 cm^−1^, were demonstrated to be carbonyl group, epoxy group and C–OH, respectively.^36^ Compared to GO, the peak intensity of rGO/CS0 around 1062, 1389 and 1730 cm^−1^ disappeared completely, indicating almost oxygen-containing groups were released from GO. And the CS characteristic peak showed the distinctive absorption band at 3440 cm^−1^ (combination of stretching of –OH and –NH). The absorption peaks at 1156 (bridge-O-stretch), 1075 and 1033 cm^−1^ (skeletal vibration involving the C–O stretching) were the characteristic peaks of CS.^37^ With the increasing content of CS, the-NH stretch peak (at 3400 cm^−1^) broadened and shifted to lower wavenumber.

#### 3.3.2 Raman spectroscopy analysis of rGO/CS*n*

Raman spectroscopy is often used to investigate various carbon composite materials.^38^ The Raman spectroscopy of the rGO/CS*n* was showed in Fig. 4(b). There were two obvious peaks at 1344 and 1576 cm^−1^, which were deemed to be the D and G bands, respectively.^39,40^ The D/G ratio represented the sp^3^/sp^2^ ratio of carbon atoms.^41^ During the oxidation process, some functional oxygen-containing groups could be inserted into graphite. Meanwhile, the oxidation process resulted in the hexagonal mesh ring structure transforming from sp^2^ to sp^3^. The destroyed original structure could lead to a decrease in the order of structure of the graphite and an increase of defects. As shown in Fig. 4(b), the ratio of the D and G bands intensity of rGO/CS*n* increased after the reduction compared with GO, indicating disordered GO sheets.^32^ The increased ratio of D/G bands intensity of rGO/CS*n* demonstrated the disappearance of the functional oxygen-containing groups and restoration of the sp^2^ network during the reduction process.^13,42^ In addition, the D/G ratio attenuated gradually with the increase of CS, probably because the CS chains used the electrostatic interaction to make the GO sheets form multilayer rGO sheets during the process of restoring self-assembly. Multilayer rGO sheets tended to orderly and made it a more complete pore wall structure, which were better for the structure of material.

#### 3.3.3 XRD analysis of rGO/CS*n*

Fig. 4(c) showed the XRD patterns of the samples. There was a sharp diffraction peak at 26.46° (002), which represented an interlayer distance of 0.34 nm indicating the highly organized layer structure of graphite.^43,44^ There was a diffraction peak at 11.4° (001), showing an interlay space of 0.78 nm (*d*-spacing) between GO sheets. The enhanced interlayer distance was due to the emerging hydroxyl, epoxy, and carboxyl groups on GO sheets.^45^ There was a diffraction peak appearing at 25.87° (001) for rGO/CS0. After reduction, the *d*-spacing of the GO attenuated from 0.78 to 0.34 nm. In addition, the X-ray diffraction pattern of CS exhibited two reflection peaks at 2*θ* = 10.2° and 19.8°, indicating the crystalline structure and amorphous structure of CS.^46,47^ It was apparently obvious that the crystalline structure of CS significantly affected by rGO sheets and the characteristic peaks related to the pure CS changed greatly.^46^ However, compared graphene, CS was still in a weak position and not enough to make GO peak move left. Also the presence of rGO nanosheets limited the mobility of CS chains into ordered crystalline structure.^26^

#### 3.3.4 XPS analysis of rGO/CS*n*

**Fig. 5:**
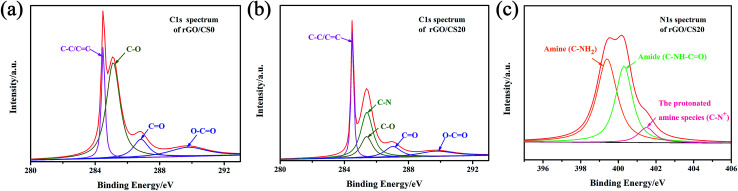
High-resolution XPS spectra of C1s for rGO/CS0 (a) and rGO/CS20 (b); high-resolution XPS spectra of N1s for rGO/CS20 (c).

The composite aerogels were investigated by XPS analysis. The C/O ratio changed from 2.35% (GO sheets) to 8.22% (rGO/CS0), indicating the loss of oxygen element during the reduction progress. There were C=C/C–C (∼284.5 eV), C–O (epoxy and hydroxyl, ∼286.5 eV), C=O (carbonyl, ∼288.3 eV) and O–C=O (carboxyl, ∼290.3 eV) groups.^48^ After the reduction, the intensities of heavily oxygenated carbon species (C–O) decreased significantly, which made the peak C=C/C–C become dominant, as see in Fig. 5(a).

The XPS peaks of C1s and N1s for the rGO/CS20 were showed in Fig. 5(b) and (c). The peak at 284.5 eV of C1s (Fig. 5(b)) was mainly due to the contribution of C–C and C=C. The other peaks at 285.4, 286.5, 288.3, 290.3 eV were ascribed to the functional groups of C–N, C–O, C=O and O–C=O, respectively.^49–51^ The arisen functional group of C–N maybe owed to the presence of the CS chains.^46,48,52,53^ The three components of N1s peak correspond to amine groups (R–NH_2_, 399.4 eV), amide (C–NH–C=O, 400.3 eV) and the protonated amine species (401.5 eV), which indicated the presence of chitosan in the composite aerogels, as shown in Fig. 5(c).^23,46^

### 3.4 BET analysis of rGO/CS*n*

**Fig. 6:**
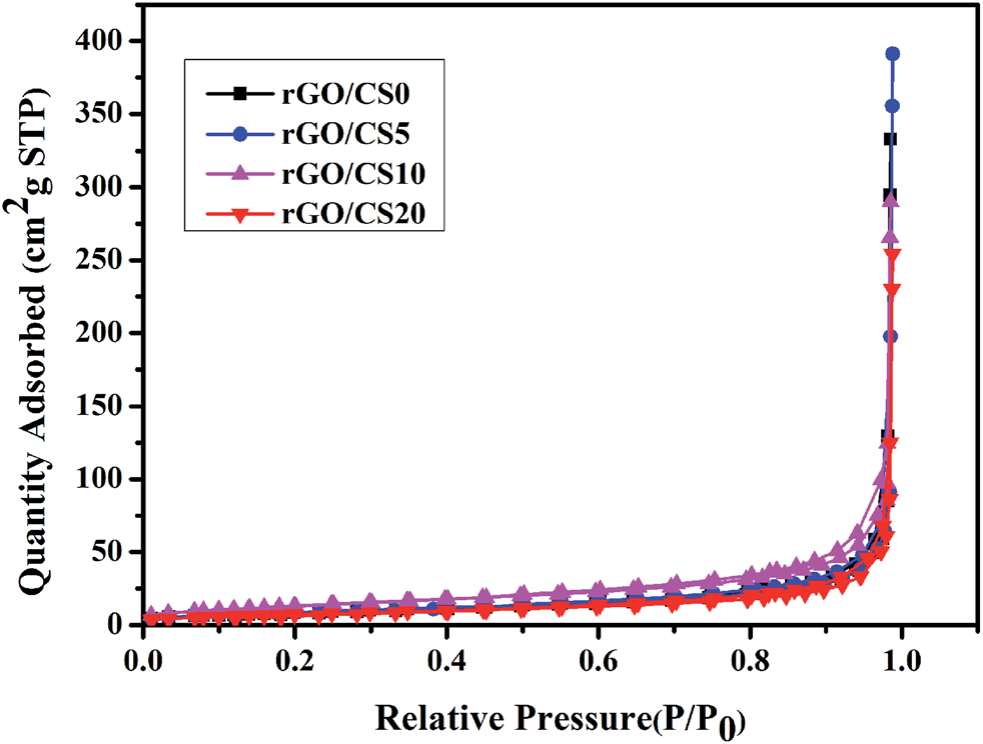
N_2_ adsorption–desorption isotherms of rGO/CS*n*.

In order to understand the porosity features of rGO/CS*n*, we performed N_2_ adsorption isotherms on it. As showed in Fig. 6, the adsorption occurring was at relative pressure smaller than 0.2 because of the micropores, and mesopores lead to the adsorption occurring at relative pressure larger than 0.8.^54^ The results showed that the exists macropore and hierarchical pores in rGO/CS*n*. Table 1 showed the specific surface area and the porosity parameters. The surface area of rGO/CS*n* was 32.38, 48.88, 32.17 and 27.05 m^2^ g^−1^ for rGO/CS0, rGO/CS5, rGO/CS10 and rGO/CS20, respectively. When the concentration of CS was increased, the specific surface area was decreased for rGO/CS*n*. The mean micropore size was 95.0861 nm, 95.2953 nm and 99.9258 nm 115.2029 nm for rGO/CS0, rGO/CS5, rGO/CS10 and rGO/CS20, respectively. The mean macropore size was gradually increased with increasing concentration of CS, because of the laminated of rGO sheets.

**Table 1:** Surface area, pore volume, and pore size of rGO/CS*n*

Samples	Surface area (m^2^ g^−1^)	Pore volume (cm^3^ g^−1^)	Mean mesopore size (nm)
rGO/CS0	32.38	0.080893	95.0861
rGO/CS5	48.88	0.116205	95.2953
rGO/CS10	32.17	0.077915	99.9258
rGO/CS20	27.05	0.076652	115.2029

### 3.5 Mechanical properties of rGO/CS*n*

#### 3.5.1 The stress–strain curve of rGO/CS*n*

**Fig. 7:**
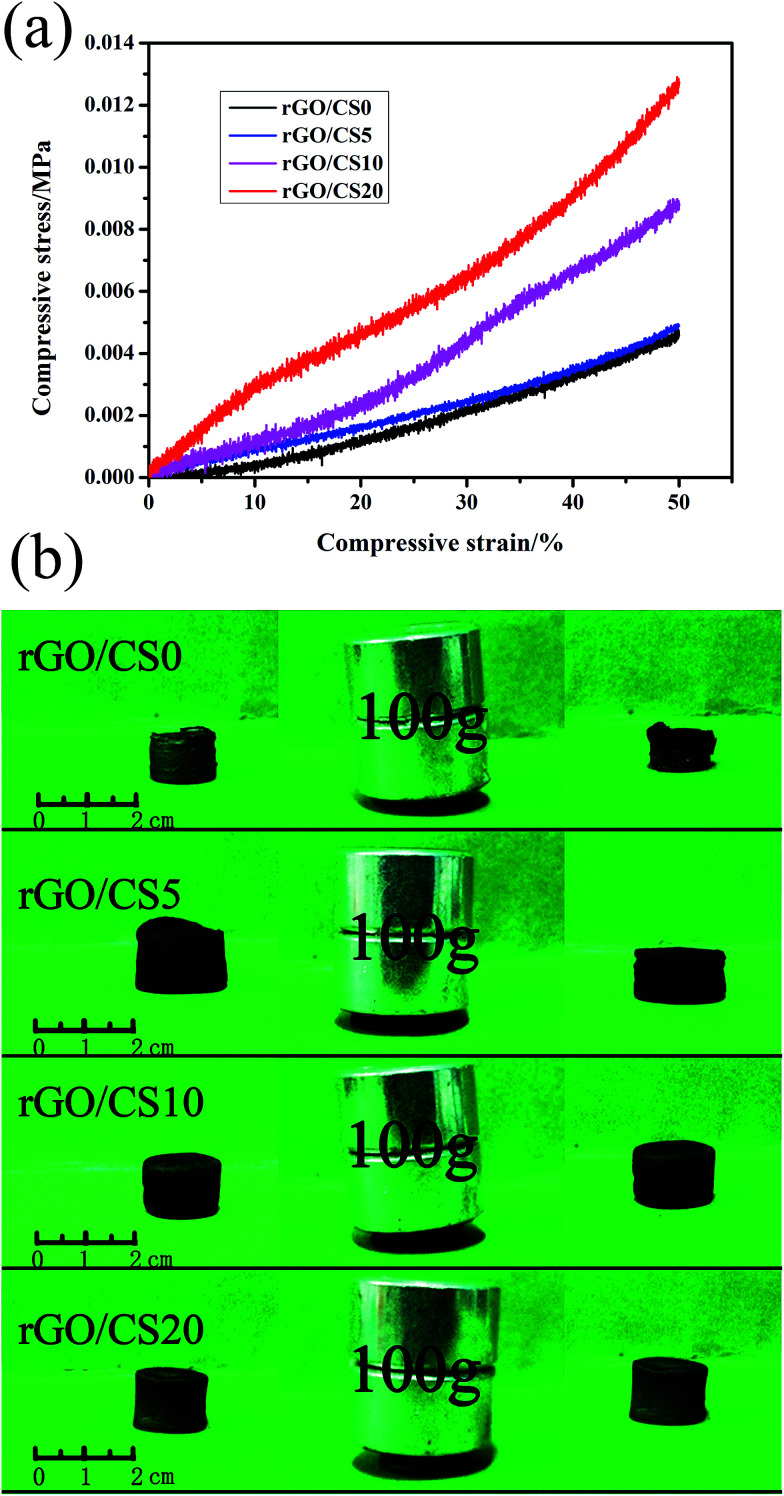
The stress–strain curves of rGO/CS*n* at the maximum strain of 50% (a); photographs of the mechanical properties of rGO/CS*n* (b).

As mentioned above, the integrity of the pore walls reflected the mechanical strength of the aerogels. Noticeably, compressive strength of rGO/CS*n* was increased with the addition of CS, as showed in Fig. 7(a). And the rGO/CS20 could support a static load at least 100 g, about 8500 times of its own weight without collapsing as showed in Fig. 7(b). After the static load, the rGO/CS0 material collapsed, the rGO/CS5 material undergone a little deformation, while the morphology of rGO/CS10 and rGO/CS20 did not change significantly. After embedding chitosan materials, the mechanical properties of rGO/CS*n* composites had been greatly enhanced. The compressive strength of rGO/CS20 was about 13 kPa and its modulus was about 26 kPa (calculated from the strain–stress curve), respectively. The improved mechanical strength was because of the CS, which could make the GO nanosheets transform to the multilayer to form complete macropore.

#### 3.5.2 Cyclic compression test of rGO/CS*n*

**Fig. 8:**
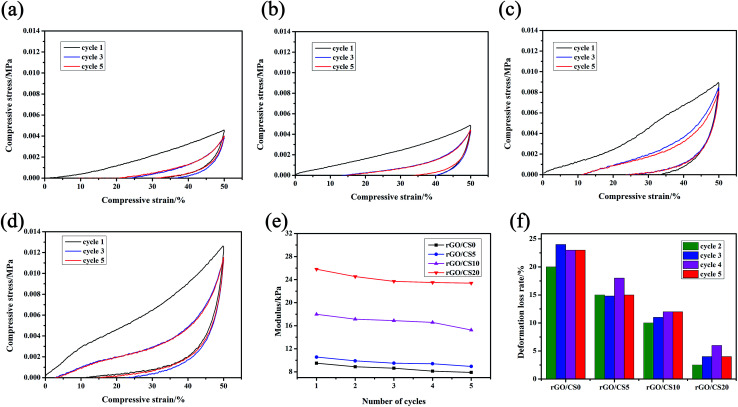
The stress–strain curves of rGO/CS*n* at the maximum strain of 50% for 5 cycles: (a) rGO/CS0, (b) rGO/CS5, (c) rGO/CS10 and (d) rGO/CS20; the Young's modulus (e) and deformation loss rate (f) for different cycles derived from.

The cyclic strain–stress curves to strain up to 50% of rGO/CS*n* were showed in Fig. 8(a)–(d). Compression tests revealed that the compressive strength and resilience of the samples were getting better with the increase content of CS in the rGO/CS*n* aerogels. For the rGO/CS*n* samples, the first compression cycle showed higher Young's modulus and maximum stress compared with the subsequent ones. The maximum compressive stress of rGO/CS20 was about 13 kPa. And the density of rGO/CS20 was 9.33 mg cm^−3^ which was comparable to metallic lattice (density of 14 mg cm^−3^)^55^ indicating a better mechanical strength when it was compared with the other rGO/CS*n* samples. The excellent performance could be due to the high strength and modulus of graphene walls of the macropores.^19^

Compared with the first one, the hysteresis loops for the third cycle shrunk and the following loading curves almost overlapped with each other. These phenomena indicated that little breakdown occurred in the cycles. And the unloading curves almost coincided (including the first cycle), suggesting that the energy stored in each cycle was invariable.^19^ After 5 cycles of compression, the maximum stress was lower 14.6% (rGO/CS0), 12.7% (rGO/CS5), 12.5% (rGO/CS10) and 8.3% (rGO/CS20) than that of the first cycle. It was because the CS made the material had a more complete pore wall built by rGO nanosheets which provided a better mechanical strength.^19,56^

Fig. 8(e) showed the Young's modulus derived from the different cycles. The maximum modulus decreased along with the cycle times of compression. The deformation loss ratio from the 2nd to the 5th cycle was showed in Fig. 8(f). The deformation loss rate reflected the strength of each material after it had been subjected to cyclic compression. After five cycles of compression, rGO/CS20 had the smallest deformation loss ratio, showing its excellent mechanical strength.

### 3.6 Adsorption of bilirubin by rGO/CS*n*

#### 3.6.1 Adsorption kinetics

**Fig. 9:**
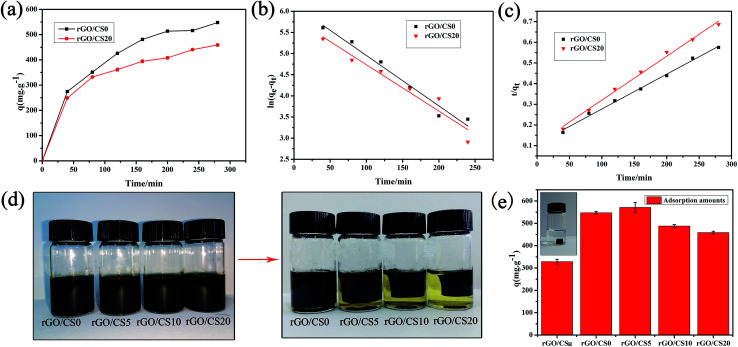
The adsorption kinetics of bilirubin by rGO/CS0 and rGO/CS20 materials (a); the pseudo-first order kinetics model (b) and pseudo-second order kinetics model (c) of the rGO/CS0 and rGO/CS20 materials; the photos of rGO/CS*n* before and after reduced by sodium ascorbate (d), and rGO/CS0 reduced by hydrothermal method (the illustration of (e)); the adsorption capacity for bilirubin by the materials (e).

Fig. 9(a) showed the adsorption kinetics of the rGO/CS0 and rGO/CS20. The adsorption reached equilibrium at 280 min. After adsorption for 40 minutes, the adsorption capacity of rGO/CS0 and rGO/CS20 for bilirubin was 274.7 and 248.8 mg g^−1^, respectively, which were much higher than that of other adsorbents.^8^ When adsorption equilibrium was reached, the adsorption efficiency was 78.2% for rGO/CS0 and 65.6% for rGO/CS20, respectively. These results illustrated that rGO was crucial in bilirubin adsorption. The adsorption of rGO for bilirubin may be due to the hydrophobic interaction between them and π–π interactions, which was the dominating force.^5,57^ The addition of CS improved the mechanical properties of the rGO/CS20 and ensured high adsorption capacity than those of previously reported adsorbents in the literature.^1,10,58–60^

The photos of rGO/CS*n* before and after reduced by sodium ascorbate were shown in Fig. 9(d). The densities of rGO/CS*n* samples were 15.06 (rGO/CS0), 5.63 (rGO/CS5), 7.02 (rGO/CS10) and 9.33 mg cm^−3^ (rGO/CS20), respectively. Also, graphene aerogels (rGO/CS_H_) reduced by hydrothermal method was shown in the illustration of Fig. 9(e) and the density of rGO/CS_H_ was 86.62 mg cm^−3^. In order to prove the superiority of adsorption performance, we compared adsorption capacity of rGO/CS_H_ with the rGO/CS*n* materials (see in Fig. 9(e)). After 280 min, the adsorption capacities of rGO/CS0, rGO/CS5, rGO/CS10 and rGO/CS20 for bilirubin were 547.6, 571.2, 487.8 and 458.9 mg g^−1^, respectively. The adsorption capacity of rGO/CS_H_ was only 328.4 mg g^−1^.

The two kinetic model was displayed in Fig. 9(b) and (c), and all the kinetic parameters were shown in Table 2. By comparing the fitting coefficient (*R*^2^), the *R*^2^ values (0.994 < *R*^2^ < 0.995) for the pseudo-second-order model were higher than *R*^2^ values (0.931 < *R*^2^ < 0.968) for the pseudo-first-order model. The pseudo-second-order model better fitted the experimental data than the pseudo-first-order one. This implied that the adsorption of rGO/CS*n* for bilirubin could better fit the pseudo-second-order model. And the calculated equilibrium adsorption capacities (*q*_e_) of the pseudo-second-order model were also more in line with the actual adsorption situation.

**Table 2:** The parameters derived from the pseudo-first order kinetic and the pseudo-second order kinetic of rGO/CS*n* materials

Samples	Experimental	Pseudo-first-order kinetic			Pseudo-second-order kinetic		
	*Q*_e_ (mg g^−1^)	*k*_1_ (g mg^−1^ min^−1^)	*R* ^2^	*q*_e_ (mg g^−1^)	*k*_2_ (g mg^−1^ min^−1^)	*R* ^2^	*q*_e_ (mg g^−1^)
rGO/CS0	547.6	0.011	0.968	466.11	2.08 × 10^−5^	0.995	666.67
rGO/CS20	458.90	0.010	0.931	340.65	3.31 × 10^−5^	0.994	529.10

#### 3.6.2 Adsorption isotherm

**Fig. 10:**
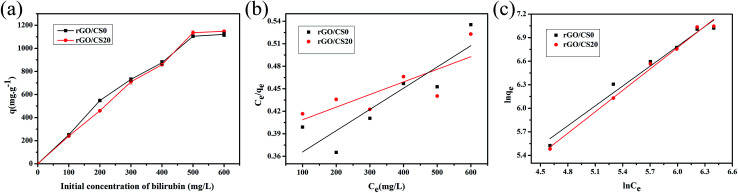
The effect of initial bilirubin concentration on adsorption capacities of rGO/CS0 and rGO/CS20 materials (a). Langmuir isotherm (b) and Freundlich isotherms (c) of bilirubin on the rGO/CS0 and rGO/CS20.

Fig. 10(a) showed the bilirubin adsorption capacities of rGO/CS0 and rGO/CS20 materials. The adsorption capacity of the rGO/CS*n* also enhanced with the initial concentration of bilirubin. And the adsorption capacity leveled off at about 500 mg L^−1^ and reached saturation. And the saturated adsorption capacity of rGO/CS0 and rGO/CS20 was 1120.7, 1147.6 mg g^−1^, respectively. The adsorption efficiency was 53.4% for rGO/CS0 and 54.6% for rGO/CS20, respectively.

The isotherm model was displayed in Fig. 10(b) and (c) and Table 3 showed the correlation coefficient for the Langmuir and Freundlich equation. We got that the adsorption of the rGO/CS*n* for bilirubin was fitted well with the Freundlich adsorption isotherm by the fitting coefficient (*R*^2^), indicating that the adsorption of rGO/CS*n* for bilirubin was a multilayer adsorption. The 1/*n* (Freundlich constant) relates to the adsorption intensity of the adsorbents. If 0.1 < 1/*n* ≤ 0.5, it is a wonderful adsorption; if 0.5 < 1/*n* ≤ 1, it is easy to adsorb; if 1/*n* > 1, it is difficult to adsorb.^61^ As we can see, the 1/*n* values of all the samples were in the range of 0.5–1, indicating that the prepared materials were good absorbents for bilirubin.^62,63^ Also it could be concluded that the rGO/CS*n* materials had a heterogeneous surface in accordance to the theoretical assumption of Freundlich model.^1^

**Table 3:** The parameters derived from the Langmuir and the Freundlich of bilirubin on rGO/CS0 and rGO/CS20 materials

Samples	Experimental	Langmuir isotherm			Freundlich isotherm		
	*Q*_e_ (mg g^−1^)	*K*_L_ (L mg^−1^)	*R* ^2^	*q*_max_ (mg g^−1^)	*K*_F_ (L g^−1^)	*R* ^2^	1/*n*
rGO/CS0	1120.7	4.29 × 10^−4^	0.550	5949.40	5.65	0.937	0.843
rGO/CS20	1147.6	8.40 × 10^−4^	0.745	3531.80	3.75	0.989	0.909

#### 3.6.3 Bilirubin adsorption in the presence of BSA

**Fig. 11:**
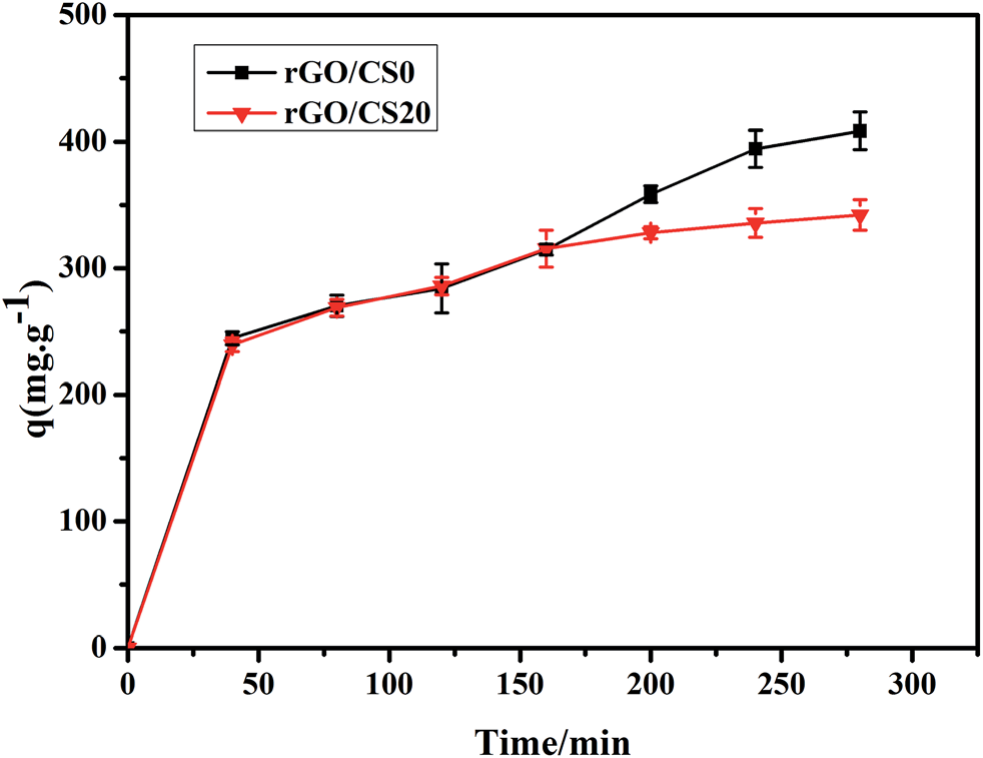
The adsorption kinetics of bilirubin/BSA (w/w = 1 : 1) by rGO/CS0 and rGO/CS20.

In blood, bilirubin strongly tends to form a complex with albumin.^1^ As showed in Fig. 11, the adsorption capacity of rGO/CS0 and rGO/CS20 decreased in the presence of BSA, but all of them could maintain the high adsorption. After 280 min of perfusion, the adsorption capacity of each material was as follows: 408.6 mg g^−1^ for rGO/CS0 and 342.3 mg g^−1^ for rGO/CS20. All adsorption capacity for bilirubin decreased about 23.4% compared with bilirubin solution without albumin. The rGO/CS*n* material can still keep good adsorption capacity at the presence of albumin, showing excellent adsorption performance.

### 3.7 Coagulation assay and hemolysis assay

**Fig. 12:**
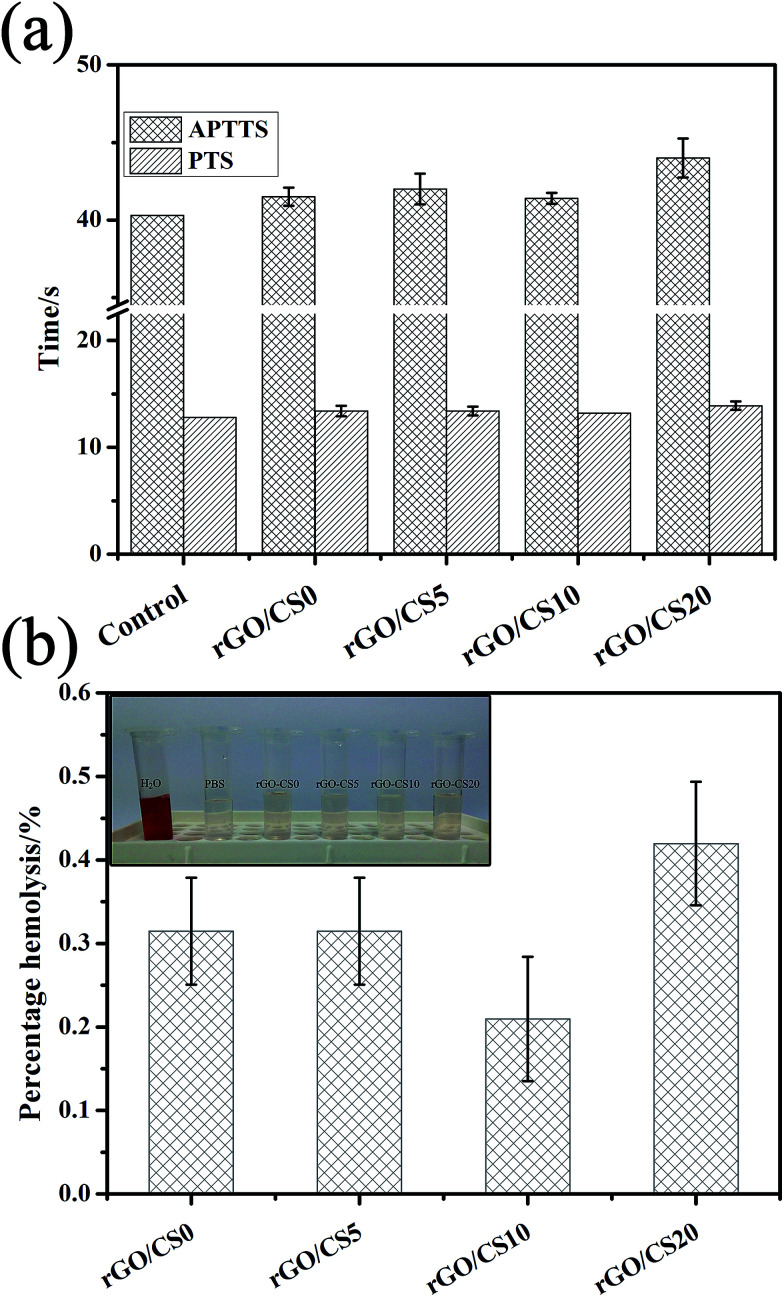
Coagulation times (a) and hemolysis (b) analysis of rGO/CS*n* materials.

APTT and PT were used to study the intrinsic-common coagulation pathway and the extrinsic-common coagulation pathway, respectively.^64^ For coagulation assay, the experimental value of the blank control group was 40.3 s for APTT and 12.8 s for PT. The value of the control was lower than that of the experimental group, as showed in the Fig. 12(a). With the increase content of CS, the coagulation effect of the material on the blood did not show regular changes. Compared to the phosphate buffer control, the value of APTT was significantly prolonged by rGO/CS20, implying the inhibition of the intrinsic coagulation pathway. The value of PT did not change significantly with the increase content of CS. All the results indicated that rGO/CS*n* materials did not elicit any significant coagulation. As showed in Fig. 12(b), the hemolysis of all the rGO/CS*n* materials was far below the accepted threshold value of 5% (GB/T 16886 and ISO 10993), indicating the good blood compatibility. The content of CS in the material did not influence the hemolysis ratio. The rGO/CS20 showed a relatively high hemolysis ratio, but the value is still lower than the national standard.

## 4. Conclusions

We have prepared rGO/CS*n* composite aerogels by a self-assembly method. To form the 3D network structure of graphene with high strength, sodium ascorbate was used as a reducing agent and embedded in CS. And the aerogel was obtained by the freeze drying. Compared with the preparation of similar materials, the proposed preparation method was simple, low cost, scalable, and green. The addition of CS not only rendered the rGO/CS*n* with good mechanical strength, but also promoted the bilirubin adsorption capacity because of the porous structure of the aerogels. The results showed a good combination of CS with graphene sheets. rGO/CS20 aerogel possessed good mechanical properties and achieved a high bilirubin adsorption capacity up to 458.9 mg g^−1^ (much higher than those of other reported adsorbents). The rGO/CS*n* with good blood compatibility and high capacity adsorption was potential candidates for blood purification.

## Conflicts of interest

There are no conflicts to declare.

## Supplementary Material
